# Effects of COVID-19 on Autism Spectrum Disorder in Qatar

**DOI:** 10.3389/fpsyt.2024.1322011

**Published:** 2024-02-20

**Authors:** Fouad A. Alshaban, Iman Ghazal, Sanaa T. Al-Harahsheh, Mustafa Lotfy, Hawraa Al-Shammari, Fatema Al-Faraj, I. Richard Thompson, Allison B. Ratto, Assal Nasir, Mohamed Tolefat

**Affiliations:** ^1^ Qatar Biomedical Research Institute, Hamad bin Khalifa University, Doha, Qatar; ^2^ College of Health and Life Sciences, Hamad Bin Khalifa University, Doha, Qatar; ^3^ World Innovation Summit for Health (WISH), Qatar Foundation, Doha, Qatar; ^4^ Community Outreach, Qatar Autism Family Association, Doha, Qatar; ^5^ Center for Autism Spectrum Disorders, Childrens National Health System, Rockville, MD, United States; ^6^ Department of Biological Sciences, Drexel University, Philadelphia, PA, United States; ^7^ Autism Department, Shafallah Center for Children with Disabilities, Doha, Qatar

**Keywords:** COVID-19, social restrictions, ASD, stress, support

## Abstract

**Introduction:**

The unprecedented impact of the coronavirus pandemic (COVID-19) has had profound implications on the ASD community, including disrupting daily life, increasing stress and emotional dysregulation in autistic children, and worsening individual and family well-being.

**Methods:**

This study used quantitative and qualitative survey data from parents in Qatar (n=271), to understand the impact of the COVID-19 pandemic on autistic children and their families in Qatar. The questionnaire was a combination of open-ended (qualitative) and closed-ended (quantitative) questions to explore patterns in the experiences of the different families, as well as to contrive themes. The survey was created in a way to evaluate the psychological, academic/intervention, economic, and other impacts of the pandemic related measures on a sample of multicultural families residing in the State of Qatar during the peak period of confinement and physical distancing in 2020. Data acquisition involved the utilization of Google Forms. Subsequent quantitative analysis employed the SPSS software and chi-square analysis for numerical examination, enabling the characterization of the studied population and exploration of associations between parental stress levels and variables such as employment status, therapy accessibility, presence of hired assistance, and alterations in their childs skills. Concurrently, qualitative data from written responses underwent thorough categorization, encompassing themes such as emotional isolation, mental or financial challenges, and difficulties in obtaining support.

**Results:**

Parents expressed distress and disturbance in their daily lives, including profound disruptions to their childrens access to treatment, education, and activities. Most parents reported deteriorations in their childrens sleep (69.4%), behavioral regulation (52.8%), and acquired skills across multiple domains (54.2%). Parents also reported decreased access to family and social support networks, as well as decreased quality of clinical and community support. Qualitative analysis of parental responses revealed that child developmental regression was an important source of parental stress.

**Discussion and conclusion:**

The greater impact of the pandemic on autistic children and their families emphasizes the need for accessible and affordable health, education, and family services to manage their special needs.

## Introduction

There is no doubt that the COVID-19 pandemic, which has rapidly swept through the world, has affected nearly all aspects of life; including the health and safety of all people, especially individuals affected by acute and chronic illnesses. Individuals with neurodevelopmental disorders such as Autism Spectrum Disorder (ASD) are no less immune to the direct and indirect impacts of this pandemic. Early data suggests that people with developmental disabilities may be even more susceptible to adverse health outcomes and death from COVID-19 (1, 2). Measures taken by most countries to control and limit the spread of COVID-19 included the closure of schools and centers providing treatment, rehabilitation, educational and training services. Nonetheless, efforts were made by most service providers to find alternative ways to compensate, even partially, for these restrictions to alleviate the great burden on families of autistic individuals. While the measures taken by countries to control and limit the spread of COVID-19 are myriad in number, they can be addressed in the following categories: impact on 1) the provision of health care services, including diagnostic, treatment, and rehabilitation services; 2) educational services; 3) autistic individuals and their families; 4) medical and intervention service providers; and 5) ASD research.

ASD is characterized by pervasive impairments in social reciprocity, communication, stereotyped behaviors, and restricted interests (3). Diagnosis of ASD requires a multidisciplinary assessment by healthcare service providers. Before the COVID-19 pandemic, centers and clinics around the world, including in Qatar, usually had a long waiting list for assessment (4). With the rise of this pandemic, many of these services were interrupted for varying periods, forcing families to postpone diagnostic evaluations, in turn negatively affecting the outcome of any intervention for the child, as earlier diagnosis is strongly associated with positive lifelong outcomes for autistic individuals (5–9). Early intervention (implemented before the age of four) is associated with gains in cognition, language, adaptive behavior, social behavior, and overall quality of life for both the child and the family (10–13).

The COVID-19 pandemic has exacerbated health inequalities and increased mental health problems on a worldwide scale. Many studies have been published recently that report increased mental health challenges across multiple study settings (e.g. USA, England, India, Lebanon, Qatar, Singapore, etc.) imposed by COVID-19 (14–17), including specifically among autistic children (18). There has also been a “silver lining” of increased access to telehealth services, which long-term may address underlying disparities in healthcare systems through the dissemination of more sustainable and equitable practices for delivering efficient mental healthcare services (19). An online survey of ASD professionals highlighted several vulnerability factors of autistic individuals in coping with the COVID-19 pandemic, including challenges related to core ASD characteristics, neuropsychological traits, executive functioning difficulties, and comorbid mental health problems all vulnerability factors reported by professionals (20). Results of initial research have indeed shown a significant negative impact of the COVID-19 pandemic on individuals with neurodevelopmental disorders such as ASD (21–23). Autistic individuals are particularly vulnerable to the disruptions caused by the COVID-19 pandemic. Lockdowns seeking to stem the spread of the pandemic led to closures of critical support institutions, such as schools, diagnostic and treatment clinics and agencies, and outpatient mental and medical healthcare, as well as support groups and organizations. Online learning, used widely to maintain access to education, has been an insufficient replacement for many autistic individuals, due to their need for specialized direct instruction and hands-on learning. One study examining the telehealth adaptation of cognitive-behavioral therapy (CBT) for anxiety in autistic children found challenges related to distractibility and reduced control of the environment, as well as decreased reciprocity and engagement in sessions (18). Attempts to provide online educational instruction for autistic students have also substantially increased demands on both educators and families to support students learning (24), often leaving families with little more than recommendations for how to provide instruction themselves (25). In addition to educational challenges, autistic individuals and their families are also impacted by reduced access to vocational training and support, home-based intervention, and access to invaluable outlets for leisure (26). Indeed, reduced independence and disruptions to routines were reported by most families of autistic children (26).

Yet the impacts of the pandemic may not be universally negative. One recent study on the psychological impact of the pandemic on autistic individuals in Spain, showed reduced psychopathology and stress and improved feeding on both caregiver-report and self-report, despite decreases in social interaction and face-to-face relationships, with particular benefits for young adults, perhaps due to reduced stressors related to these external relationships and demands (27). In contrast, caregivers reported increased levels of stress and anxiety for themselves. The contrast between the experiences of autistic individuals and those of their caregivers in this study may be at least partially attributable to the already high-stress levels for family members of autistic individuals before the pandemic (28–30). A study based in Saudi Arabia found that parents of autistic children reported increased stress and anxiety related to caring for their autistic children during the COVID-19 pandemic (31). Parents of younger children, and mothers in particular, reported the highest levels of anxiety, and parental anxiety and mental health together predicted perceived mental health needs. Parents of autistic children may be particularly vulnerable to the effects of the COVID-19 pandemic and associated lockdowns, to their pre-existing high-stress levels which make the added stresses faced by all parents (e.g., unemployment, working from home, caring for typically developing children engaging in online learning, reduced access to social supports) even more impactful.

The objective of the present study is to evaluate the effect of measures taken to control COVID-19 pandemic on the daily functioning and well-being of autistic individuals and their families in Qatar, a country that has made critical gains in recent decades in expanding access to ASD diagnosis and treatment. Specifically, this study investigates parent-reported impacts of COVID-19 mitigation measures (i.e., lockdowns restricting access to services and requiring families to remain at home) on the daily functioning of autistic children, access to services, and parent psychological well-being. We hypothesized that parental stress would be significantly associated with parent employment status, access to in-home therapy, access to specialized intervention, presence of hired help in the home (e.g., housekeeper, nanny, etc.), and regression in child skills. Of note, hired help in the home is relatively common in Qatar and these untrained individuals often provide substantial support to families in caring for autistic children in the home. Thus, this variable was important to include in this specific cultural context. The qualitative data not only allowed us to add more context to the individual situation of each family and provided an open view of the differences in experiences during that time but also played a crucial role in strengthening our study design and findings. By considering the qualitative data questions, we were able to gain more perspective and contrive additional information about the common and uncommon challenges parents faced during this period. This holistic approach, combining qualitative and quantitative data, contributes to a comprehensive understanding of the effects of COVID-19 mitigation measures on the daily functioning and well-being of autistic individuals and their families in Qatar. Examining the impact of COVID-19 on autistic children in Qatar is particularly vital, as it allows for a nuanced understanding of how cultural and contextual factors unique to the region may shape the experiences of families and individuals with autism.

## Materials and methods

### Study design

To examine and evaluate the effect of measures taken to control the COVID-19 pandemic on autistic individuals and their families, a mixed-method approach (32), collecting quantitative and qualitative survey data from parents of autistic children in Qatar was used. To collect the data, we created a questionnaire to be completed through Google Forms. The survey took place between September and December of 2020. The Participants gave their informed consent via the online platform or by email. This study was approved by Qatar Biomedical Research Institute Institutional Review Board (IRB) research ethics committee. All procedures performed were in accordance with the ethical standards of the institutional and/or national research committee and with the 1964 Helsinki declaration and its later amendments or comparable ethical standards.

### Participants

A convenience sample of 271 parents who self-identified as having at least one child aged between 3 and 18 years with a formal ASD diagnosis was used. All families resided in the State of Qatar during the COVID-19 pandemic. Participants were recruited via online advertisement through various social media channels, organizations (e.g., Qatar Autism Society) and via existing databases of autistic individuals (33) that have previously given their consent to be contacted for ASD-related research. We contacted parents of children with the diagnosis of ASD by telephone or by e-mail (online form). Those with more than one autistic child were asked to focus on just one child (of their own choosing) in their responses.

### Instruments

Google Forms was used to create an online parent survey to be shared through the dissemination of a hyperlink. All participants provided electronic informed consent that contained information about the purpose of the study, procedures, benefits of participating, voluntary participation, and contact information of the researchers. Since no questionnaire existed for evaluating the target concept, the researchers in this study created one. All the survey questions were developed based on the available literature in the subject matter e.g. The Autism Parenting Stress Index (APSI) and the World Health Organizations Quality of Life Questionnaire with Parents of Children with Autistic Disorder (34, 35). The survey questions were not contrived directly from these questionnaires, they were rather used as a guide as to how to design our survey and which questions to ask. The questionnaire was pilot tested first on a sample of three parents of autistic children to assess the studys measures for reliability, and to establish validity before embarking on the full study. Parents were asked to evaluate appropriateness of response options, time taken to complete it, and clarity of the questions in terms of language, wording, and meaning. Necessary corrections were considered and made by authors as appropriate. All measures and informed consent were then translated from English to Arabic by bilingual researchers in this study to enable families to complete forms in their preferred language [N=58 (21.4%) English, N=213 (78.6%) Arabic]. The final version of the questionnaire was comprised of 36 multiple-choice and open-ended questions about sociodemographic information, the impact of the pandemic on autistic childrens daily life, access to information and services/support during the pandemic period, parents pandemic experience and primary challenges, and parents overall health status and stress levels.

### Procedures

The preliminary version of the questionnaire was revised in an iterative process by three of the authors (FA, SA, and IG). All the researchers involved in data collection are Arabs fluent in both English and Arabic who have extensive experience in ASD research within the Arab community. The study employed two distinct recruitment methods. Initially, participants were reached through email, wherein they received a survey link. Families who chose to engage accessed, completed, and submitted the survey electronically. Alternatively, a second recruitment mode involved contacting families via phone. During this outreach, the studys purpose was explained, and families were given the option to either complete the survey over the phone with the researcher or independently. Consent was obtained electronically through an online form in all instances. Notably, this approach was particularly relevant given the studys timeframe coinciding with social distancing measures and the closure of centers, schools, and clinics. The survey took approximately 10- 15 minutes to fill out. If a parent had more than one autistic child, a separate survey was filled for each child. The survey pertained to the COVID-19 pandemic, and more specifically, the period of confinement and physical distancing in 2020.

### Data analysis

The final raw data were downloaded from Google Forms into a Microsoft Excel file for analysis using SPSS software (Version 26.0). For the quantitative data, descriptive statistics were used to characterize the sample and examine survey responses. Then, chi square and likelihood ratio tests were used to investigate the association between parents stress levels and employment status, access to in-home therapy, access to any sort of specialized intervention, presence of hired help in the home (e.g., housekeeper, nanny, etc.), and regression in child skills. In order to identify the challenges that parents with autistic children face during the pandemic, the qualitative data collected by written responses through semi-structured forms was analyzed using thematic content analysis technique by identifying themes and subthemes and associating them with examples (36). Three authors (FA, SA, and IG) independently evaluated responses and placed them into categories (e.g., social isolation, mental and psychological problems, financial issues, access to regular support, service interruption, etc.). In the rare instances of discrepant category attribution, the authors reached consensus through discussion.

## Results

### Sociodemographic information

Parents provided basic sociodemographic information about themselves and their autistic child, including parent gender, nationality, and work status, as well as child age, gender, and diagnoses, along with the number of children at home and membership in ASD advocacy/family support networks. The majority of the participants were female (mothers; 66.4%), and non-Qatari (59.4%). Almost all fathers (90.4%) were employed, while most mothers (53.9%) were unemployed. The majority of parents do not work from home (64.9%) and are members of ASD advocacy and family support networks (67.2%). An average of four children lived at the home, including autistic children. The vast majority of autistic children were male (79.0%; n=214) with most (62.7%) falling between the ages of 9 and 13. Approximately one-third (31.0%) of the children had co-occurring conditions such as ADHD or epilepsy (see **Table 1** for more details). To ensure a comprehensive understanding of each participants current level of functioning, our survey questions were occasionally supplemented with sub-questions. Some questions offered multiple options for selection, followed by inquiries about the presence of comorbid conditions, utilizing a yes/no format. Participants selecting “yes” were then prompted to specify the particular condition. Notably, information regarding the childs verbal ability was not explicitly addressed in a dedicated question; instead, it relied on data reported in the comorbid conditions or additional comments sections of relevant survey questions.

**Table 1:** Sociodemographic information of autistic children and their parents.

Variable	N (Total N=271)	%
Parent gender		
Female	187	69.0%
Parent nationality (Qatari v. Non-Qatari)		
Qatari	110	40.6%
Parental employment status		
Employed fathers	245	90.4%
Employed mothers	125	46.1%
Employed parent currently working from home	95	35.1%
*Member of ASD advocacy/support group*	182	67.2%
Child gender		
Male	214	79.0%
Child age range		
3 to 8	81	29.9%
9 to 13	170	62.7%
14 to 18	12	4.4%
above 18	8	3.0%
Child ASD diagnosis		
ASD	260	95.9%
ASD: High Functioning	4	1.5%
ASD and Down syndrome	4	1.5%
ASD: Nonverbal	3	1.1%
*Child co-occurring conditions present*	84	31.0%

### Survey responses: quantitative analyses of survey results

#### Impact of COVID-19 on autistic children and their families

Parent survey responses indicated that the COVID-19 pandemic has had a significant on autistic children in Qatar. As seen in **Table 2**, the majority of parents reported that their childrens sleeping schedules had been disrupted or changed since the start of home quarantine (69.4%). Most reported that their children displayed new problem behaviors (52.8%), such as hyperactivity, tantrums, self-injurious behavior, new stereotyped behaviors, and/or disrupted sleep. Most parents also endorsed a noticeable regression in their childrens previously gained skills (54.2%), such as difficulty following instructions, reduced self-help skills, worsening social skills, increased attention issues, and/or reductions in communication and academic skills. Furthermore, most parents (64.6%) attributed specific problems/issues to the challenges of being home all day (e.g., boredom, aggression, mood/irritability issues, etc.). However, the vast majority of parents (73.1%) indicated that their children were still participating in physical activities, either inside or outside the home. The COVID-19 pandemic was also found to have a significant impact on the daily lives of families in this study. The vast majority of parents (97.0%) reported that COVID-19 caused substantial changes in their daily routine, reduced social contact (96.7%), and increased their stress levels (58.7%).

**Table 2:** Impact of COVID-19 autistic children and their families.

Survey Question	Yes (N)	% Yes
Is your child practicing any physical activity inside the home or outdoors?	198	73.1%
Did your childs sleeping schedule change since the start of the home quarantine/physical distancing measures?	188	69.4%
Did your child exhibit any negative behaviors since the start of the home quarantine/physical distancing measures?	143	52.8%
Did you face any problems/issues with your child being at home all day?	175	64.6%
Since the start of the home quarantine/physical distancing measures, did you notice any regression in your childs previously gained skills?	147	54.2%
Has your family been reducing social contact as a result of COVID19?	262	96.7%
Has COVID-19 led to significant changes in your familys daily routine?	263	97.0%
Survey Question	N	%
On a scale of 1-5, how would you rate your stress level during this time:		
Not stressed at all Normal level of stress Neutral Stressed Very stressed	16 50 46 85 74	5.9% 18.5% 17.0% 31.4% 27.3%

Chi square tests were then used to assess the relationship between parental stress levels and key variables of interest, including: parent employment status, access to in-home therapy, access to any sort of specialized intervention, presence of hired help in the home (e.g., housekeeper, nanny, etc.), and regression in child skills. Findings indicated that from the predicted variables (**Table 3**), parent stress was significantly associated only with a regression in the childs previously gained skills, such that child skill regression was associated with increased parental stress [χ^2^ (4, N=271) = 12.580, *p*=.014), such that child regression increased the likelihood of high parental stress thirteen-fold (Likelihood Ratio=13.127).

**Table 3:** Summary results of Chi square and likelihood ratio tests of predictors with parent stress level.

Variable	Pearsons χ^2^	Likelihood Ratio
Father employment status	1.441 (4, N=271; *p*=.837)	1.501
Mother employment status	4.858 (4, N=271; *p*=.302)	4.903
Child receiving in-home therapy	11.972 (4, N=271; *p*=.152)	14.446
Child receiving any specialized intervention	7.905 (4, N=271; *p*=.443)	7.720
Presence of hired help in the home	11.522 (4, N=271; *p*=.485)	11.236
Regression in child skills.	12.580 (4, N=271; *p*=.014)	13.127

#### Impact of COVID-19 on *accessing* and *receiving services*/*support*

As shown in **Table 4**, the majority of parents in this study stated that they had difficulty receiving support from extended families/friends/community during the pandemic (57.6%), that they could not access usual support due to COVID-19 (63.1%), that they were only partially or not at all supported by their families and community (55%), and that they did not have access to any online ASD support (e.g. family support groups, therapeutic support) (63.5%). Most also reported that their autistic children were not participating in online learning (58.7%) and did not receive any intervention sessions from a qualified specialist during the home quarantine period (76.0%).

**Table 4:** The impact of COVID-19 on accessing and receiving services/support.

Survey Question	Yes (N)	% Yes
Did you find difficulty receiving support from your extended family/friends/community during this time?	156	57.6%
Is your autistic child presently participating in online learning?	112	41.3%
Is your child receiving any intervention sessions from a qualified specialist during the period of the home quarantine/physical distancing measures?	65	24.0%
Does your family presently have access to any online ASD support (e.g., family support groups, therapeutic support, etc.)?	99	36.5%
Is there any type of support that your family usually receives but cannot access in the meantime because of COVID-19?	171	63.1%
Survey Question	N	%
On a scale of 1-4, how supported do you feel, whether from other family members, the community, etc.		
Not supported at all Slightly supported Supported Very supported	65 84 76 46	24.0% 31.0% 28.0% 17.0%

### Qualitative analyses: the main challenges families face during the pandemic

Parents opinions regarding the main challenges they faced during the pandemic were analyzed with thematic content analysis (**Table 5**). The main challenges that parents face during the pandemic are grouped under social isolation, mental health/psychological problems, access to regular support, service interruption, financial, and job challenges, increase in childs negative behaviors and skill regression, outdoor activities, and learning/education challenges. The highest frequency is in the mental health/psychological problems, increase in childs negative behaviors and skills regression categories, social isolation, and lack of outdoor activities. The lowest frequency is in the Financial and job challenges category. The sub-categories with the highest frequency are regression in gained skills (*n* =56), fear of getting the virus (*n* =30), lack of outdoor activities (*n* =20) and restricted all social outings and interaction (*n* = 20).

**Table 5:** The main challenges families face during the pandemic.

Theme	Subthemes	*n*	Quotations
**1. Social isolation**	Abrupt Social Change and social anxiety Restricted all social outing and interaction Total	15 20 35 (13.5%)	*“ The mandatory home quarantine has protected my family; however, it has also caused isolation and social anxiety in my autistic son.”* *“Social isolation was difficult for the whole family.”* *“City closure and quarantine restricted or diminished all social outings and opportunities for children to go out, interact, exert energy.”* *“The social isolation affected us all negatively as a family.”*
**2. Mental health/psychological problems**	Anxiety Depression Family Stress Fear of getting the virus Health and psychological impact on children Feeling negative Total	10 5 10 30 10 3 68 (26.3%)	*“I became anxious about cleanliness, not seeing our families.”* **“** *Fear of regression and integrating back into society and social life after quarantine.”* “*Being at home all day with the kids was difficult for me … and them.”* *“The pandemic has added more stress to an already stressful situation. Autistic children have daily routines when that routine is disrupted it has a mental and physical effect on the families and on the autistic child. … We have been lockdown since March no one has bothered to ask the already struggling familys how to cope with the lockdown.”* *“The online learning is a struggle, and the interruption of all additional therapies that were supporting my sons progress has caused a real stress and regression on our family and on his skills.”* *“Interruption of therapy and negative effect on my child, and caused high stress in our home.”* *“My wife and I are both frontline healthcare workers, this has made it very difficult physically and psychologically straining and caused anxiety as we were worried that we would bring the virus home or catch the virus and then we would have to be quarantined away from our child. This is especially difficult for expat families who have no one here.”* *“Emotional and psychological effects of the quarantine (depression, anxiety, fear for catching the virus), fear that the autistic child contracts the virus, how he would be able to handle self-quarantine (it would be impossible).”* *“Scared my kid would get the virus, who would take care of him if I had COVID: I am a single Mom and dont have family here.”* *“Fear of getting the virus and fear that if mother or father get the virus, who would take care of the autistic child during the time of quarantine or probable serious health complications.”* *“This epidemic has negative effects on the students psyche. Children with special needs do not understand quarantine and not go out, do not like to sit at home. The school was an outlet for them.”* *“The usual stress out because we cant see family and scared for them.”* “*Restricted movement and mental stress.”* *“My child and wife went to Egypt before the COVID-19 spread in Qatar, they couldnt return and now he had to be registered in a center in Egypt while they wait for the return, and it is not safe there in terms of prevention measures, and also my sons condition is much worse now because of this big change. This time has been very challenging for us.”* *“Job stress, interruption of everything”* *“Paralyzed our life and put us in really threatening issues”*
**3. Accessing to regular support**	Restricted Access to Therapeutic support and medical care Loss of support from the center and family Total	4 12 16 (6.2%)	*“Difficulty accessing Therapeutic support, medical care when we needed, and overall support from friends and community as we had been quarantined.”* *“Doctors appointments stopped, and we didnt know when they will return.”* *“Loss of support from the center and family”.*
**4. Services interruption**	Change in family daily routine and activities Interruption of intervention services Total	*12* 20 22 (8.5%)	*“ The interruption and complete change of our daily activities and routines this period has affected our childs progress very negatively* *Overall got worse in everything”.* *“Everything in the ASD service provision is profit based, everything is expensive, everything is a “show”, it is so hard to find quality intervention, the change in the daily routine affected my child greatly”* *“There was a definite negative effect from the stop of intervention”* *“The interruption of all activities and therapies that I was doing for my child to develop her social and communication skills. the interruption of services and social gatherings to me is considered the most negative aspect of COVID-19 time”.*
**5. Financial and job challenges**	Financial strain Fear for job loss add stressors Total	5 6 11 (4.2%)	*“My wife lost her job during this time, so this was one of the added stresses for us as a family, the financial strain and fear for job loss for me as well. this was definitely a stressful time for everyone”.* *“All life and daily routine changes associated with the quarantine and how COVID-19 affected our lives was a challenge and continues to be a challenge. Fear for job loss is also a challenge”.* *“Worry about if our son worsens (due to the social isolation) and also the stress due to the financial impact of COVID-19 on all jobs in the country”.* *“All measures taken were in our favor to protect our families and residents in Qatar. The only negative aspects are the financial strains and stress from fear job loss as this has affected the economy”.*
**6. Increase in childs negative behaviors and skills regression**	Regression in gained skills Behavioral challenges Total	56 10 66 (25.5%)	*“Challenge to convince a child of why they cant go out, left alone a child with communication issues. Intervention stopped and child regressed.”* *“Fear of child regressing.”* *“It was a big struggle for me not being able to deal with my childs behavior and feeling that I cant help continuing the intervention at home.”* *“Regression in skills due to stop of intervention, trying to give them useful activities to do, stress, social isolation, lack of leisure activities.”* *“Communication and behavioral regression in my child as a result of being home all day and stopping of intervention, social isolation, lack of leisure or extracurricular activities.”*
**7. Outdoor activities**	Lack of outdoor and leisure activities Travelling restriction Total	20 7 27 (10.4%)	*“Lack of social outings, leisure activities, and social interaction due to the quarantine.”* *“Closure of everything, no leisure activity, not even beaches or parks, everything was closed, it was hard, and we werent able to take him out to exert some energy.”* *“Social isolation, not being able to travel”*
**8. Learning/Education challenges**	Lack of trust on online learning School closure & regression in education Lack of educational programs for special needs and extracurricular Total	3 *6* *5* 14 (5.4%)	*“The online learning is not working out for us. It is not enough for our child and this type of learning isnt for all learners”.* *“Regression in education and daily skills”* *“Fear of lowering his school level”.* *Stopped extracurricular activities, school closures, social isolation, etc”*

## Discussion

This study used parent report surveys to gain insight into the impacts of the COVID-19 pandemic on the lives of autistic children and their families in the State of Qatar. As expected, parents reported high levels of distress and disruption to their daily lives, as has been widely reported throughout the world. The majority of parents reported that their childrens access to activities, education, and treatment was either substantially reduced or non-existent. Parents also reported reduced access to family and social support networks during this critical time of increased stress. Interestingly, parental stress was not significantly associated with support factors, such as access to treatment and social support, nor was it associated with parental employment status. This is in direct contrast to prior studies of families caring for autistic children, which routinely find an association between familial stress and access to support services (29, 37). It is possible that families were under so much stress during this intense period of lockdown that even access to support provided minimal relief.

The data also indicate that families had minimal access to support and that available supports were routinely of lower quality than those provided before the COVID-19 pandemic; thus, the lack of association indicates that the services provided were insufficient to impact family stress levels. Parental stress was significantly associated with child developmental regression, indicating that children whose support needs increased even more during this time due to skill loss likely increased parental stress. Furthermore, the majority of parents indicated that their childrens sleeping habits had been interrupted or changed since the start of home quarantine, that their children had shown negative behaviors, and that there had been a clear regression in the childrens previously acquired abilities. Beyond the quantitative data collected in this study, the qualitative data analysis provides valuable additional insights into the unique experience of parenting an autistic child during the COVID-19 pandemic. While many of the experiences endorsed by parents in quantitative data were likely shared by parents of neuro-typical children (e.g., reduced access to activities, financial and employment stress, lack of connection to support networks) (38), the qualitative data reveal stressors and experiences unique to parents of autistic children (39). Throughout the major themes that emerged, two major underlying threads were clear that connected the stress expressed in each of the themes and subthemes.

Firstly, parents emphasized that they had already been under enormous stress with insufficient support to care for their children prior to the start of the COVID-19 pandemic and subsequent lockdowns. Parents indicated that services for ASD were already scarce and that there was little societal understanding and support for the challenges that they and their children faced before the pandemic. Parents in this study shared feeling “forgotten” by society, stating that because their needs were already overlooked and their children ignored before the COVID-19 pandemic, it felt clear to them that the unique impact of the pandemic on their children and families was not recognized or considered by society at large. Thus, while they reported concerns that are similar to those echoed by parents of neuro-typical children, these concerns emerged in the context of pre-existing distress and unmet support needs. The lockdown was often framed in their comments as a new layer of stress that felt “impossible” to handle on top of their pre-existing experiences.

Secondly, parents highlighted how their childs symptoms of ASD compounded the stressors of the COVID-19 pandemic lockdown experienced by all families. Although all families experienced stressful disruptions to their daily routines, parents in this study noted that their children relied intensively on predictable routines before the pandemic, even more so than their peers did, with even small changes having the potential to be highly disruptive. These families thus felt the catastrophic changes wrought by the COVID-19 pandemic even more intensely. Similarly, families around the world were negatively impacted by the lack of opportunities for social interaction for their children. The lack of social interaction was particularly worrisome to families in this study, as difficulties with social communication and engagement skills are a core feature of ASD, which are targeted directly in treatment and education for these children. Families expressed their worries that the social isolation of the pandemic would lead to further delays in their children and/or loss of skills. Concerns about regression, which occurs much more commonly in autistic children than in neuro-typical children, also emerged across themes. Parents highlighted that not only was their child failing to learn and gain new skills during this time, but also that there was a very real risk that their child might lose previously gained skills (and in some cases had already). In addition to developmental regression and skill loss, parents also shared that their children experienced exacerbations in pre-existing negative behaviors or developed new negative behaviors, as a unique expression of the impact of the lockdowns on autistic children.

Finally, their childrens underlying difficulties with learning and social attention attempted at online education and intervention, when available, extremely difficult, or even impossible to access. Since these learners require different types of instruction, they were often left with recommendations for instruction to be practiced by the families, rather than direct instruction. The online learning format substantially increased demands on both educators and families to support students learning around the world (24), with minimal time and resources to adapt special education curriculums to online learning with the unique needs of autistic students in mind.

An additional challenge not captured by the present survey is the delay in diagnosis and identification. In addition to the impact on treatment and educational services, diagnostic assessments have also been affected, causing delays in diagnosis for many individuals. This in turn can have severe negative long-term outcomes due to delays in access to treatments and appropriate interventions (9). Access to early diagnosis and intervention is a strong predictor of long-term outcomes (40, 41), meaning that children whose diagnosis and access to treatment are delayed during this time are at substantial risk for even more negative long-term outcomes with higher support needs.

### Limitations

The current study also has some limitations. First, like many online research studies, our sample may reflect some degree of selection bias. In particular, we expect certain underestimations of extreme cases, that is, people who were either minimally or very affected by the pandemic. Second, the results of this study are limited to the immediate experience at the onset of the pandemic and, therefore, to the short-term effects of the pandemic. Future studies also need to consider the long-term effects of the pandemic as it is becoming increasingly clear that this pandemic and any future pandemic are likely to have a prolonged course. Thirdly, the study methodology and data exclusively involve input from parents/guardians, resulting in the absence of perspectives from autistic individuals themselves. This constitutes an additional limitation to the study that should be taken into account in future research.

## Conclusions

The COVID-19 pandemic and recurrent lockdowns have already had an enormous impact on our world, and their effects will be felt for years to come. As vaccinations continue to become more available and access to services and education steadily increase, we cannot simply “go back to normal.” It will be critical for providers and educators to consider the effects of the ongoing pandemic on childrens development, academic progress, and emotional well-being. Moreover, the medical community should learn from this crisis and to support autistic families in navigating burdensome times to enable autistic children to thrive. As evidenced by the high levels of distress described by parents in this study, the development of personalized formulation-based-psycho-social interventions to support and engage autistic individuals and their caregivers to help cope with the consequences of the pandemic and similar waves in the future is critical (20). As highlighted in this study, there have been substantial disruptions to the lives and developmental progress of autistic children. These disruptions will have lasting effects on their skills, including potential skill loss. Adjustments will need to be made to educational, treatment planning that adjust developmental, and educational goals to remediate skills and begin helping children catch up on lost time. The emotional impacts of this time will also need to be considered, including that many children may have had a traumatic reaction to the disruptions in their routines and lives. Trauma-informed approaches to both treatment and education are likely to have much more relevance in the field of ASD going forward than they have in the past.

There will be a pressing need for society to not only stop neglecting the needs of autistic children and their families but to begin actively supporting them in enhanced ways. Local and national governments will need to consider increased supportive initiatives for psychological support, financial support, training workshops, and online sessions for families and providers. This should also include additional support and incentives for provider and teacher training in ASD. The interruption of service provision whether home-, school-, or center-based, by experienced and highly skilled multi-disciplinary professionals is expected to have a negative effect on these service providers as they have acquired their skills and continue to enhance them through their daily practices while working with autistic individuals. This has also limited opportunities for trainees to develop critical skills needed for working in ASD. Reduced training opportunities will in turn mean fewer qualified providers are available, in a system that is already unable to fully meet the needs of autistic children. Increasing access to services through support to providers will also be critical. Moving forward into the “new normal,” governments around the world will need to make substantial and lasting investments in training, family support, education, and services for this vulnerable group of children and their families. The greater impact of the pandemic on the autistic children and their families emphasizes the need for accessible and affordable (continued) health, education, and family services to manage their special and immediate needs.

## Data availability statement

The original contributions presented in the study are included in the article/**Supplementary Material**. Further inquiries can be directed to the corresponding author.

## Ethics statement

The studies involving humans were approved by Qatar Biomedical Research Institute - Institutional Review Board (IRB). The studies were conducted in accordance with the local legislation and institutional requirements. Written informed consent for participation in this study was provided by the participants legal guardians/next of kin. The parents/legal guardians of the adult participants provided written informed consent as they are usually the ones who provide consent on their behalf.

## Author contributions

FA: Conceptualization, Data curation, Formal analysis, Funding acquisition, Investigation, Methodology, Project administration, Resources, Supervision, Writing – original draft, Writing – review & editing. IG: Data curation, Methodology, Project administration, Writing – original draft, Writing – review & editing. SA-H: Conceptualization, Formal analysis, Funding acquisition, Writing – review & editing. ML: Conceptualization, Investigation, Methodology, Writing – review & editing. HA-S: Data curation, Investigation, Writing – review & editing. FA-F: Data curation, Writing – original draft, Writing – review & editing. IT: Data curation, Formal analysis, Writing – review & editing. AR: Data curation, Formal analysis, Writing – review & editing. AN: Writing – review & editing. MT: Conceptualization, Investigation, Resources, Writing – review & editing.
