# Supplementary material for: Effects of COVID-19 on Autism Spectrum Disorder in Qatar
Source: Front Psychiatry. 2024 Feb 20;15:1322011. doi: 10.3389/fpsyt.2024.1322011 (PMC10913057; doi:10.3389/fpsyt.2024.1322011)
Supplement: Supplementary file 1 [file DataSheet_1.pdf]

Appendix 1: Research's survey/questionnaire (any question with an asterisk (*) had to be filled for successful submission of the filled survey

SURVEY TO MEASURE THE EFFECT OF COVID-19 HOME QUARANTINE/PHYSICAL DISTANCING MEASURES ON AUTISTIC INDIVIDUALS AND THEIR FAMILIES

INTRODUCTION

We invite you to take part in a research study called "The Effect of the Coronavirus Disease (COVID-19) Pandemic on Autism Spectrum Disorder (ASD)". You were selected as a possible participant in this study because you fall within the inclusion criteria of our research project; a resident in the State of Qatar (Qatari National or expatriate, who has one or more than one autistic child). Please take your time to read this form, ask any questions you may have and make your decision. We encourage you to discuss your decision with your family, friends and doctor(s).

WHAT IS THE PURPOSE OF THIS STUDY?

This study is being done to evaluate of effect of measures taken to control COVID-19 pandemic on services provided for autistic individuals and their families. It will also explore the measures taken by service providers to find alternative ways to make their services available for those individuals. This research project will help to evaluate the efficiency of service provision in different health care settings, and finding the best way of delivering these services to individuals with special needs during the time of an unforeseen crisis. The outcome of this research will help to find alternative ways to help autistic individuals in getting the best ways possible in delivering health care and other services to them and their families, in case we face a similar situation in the future.

WHAT ELSE SHOULD I KNOW ABOUT THIS RESEARCH STUDY?

It is important that you read and understand several points that apply to all who take part in our studies:

- Taking part in the study is entirely voluntary and refusal to participate will not affect any rights or benefits you normally have;
- You may or may not benefit from taking part in the study, but knowledge may be gained from your participation that may help others; and
- You may stop being in the study at any time without any penalty or losing any of the benefits you would have normally received.
- The nature of the study, the benefits, risks, discomforts and other information about the study are discussed further below. If any new information is learned, at any time during the research, which might affect your participation in the study, we will tell you. We urge you to ask any questions you have about this study with the staff members who will explain it to you and with your own advisors prior to agreeing to participate.

WHAT HAPPENS IF I AGREE TO BE IN THE STUDY?

- If you agree to participate in this study, we will obtain demographic and clinical information from you regarding all immediate family members, including brothers and sisters. (Your name will not be taken and your information will be anonymized).
- Completion of a survey either online or by phone (to be completed with one of our research assistants). The survey includes 41 questions developed to capture the psychological, economic, personal, academic, and clinical impact of the current situation on individuals of ASD and their families living in Qatar (both Qataris and non-Qataris).

WHAT ARE THE RISKS AND SIDE EFFECTS OF THIS STUDY?

There are no physical risks associated with the participation in the study, since the study only request you to answer a set of questionnaires.

ARE THERE ANY BENEFITS TO TAKING PART IN THE STUDY?

This study is not designed to provide direct benefits to any participants.
You may or may not get any direct benefit from being in this study. We hope the information learned from this study will benefit you or others in the future.

WHAT ABOUT CONFIDENTIALITY?

Since the survey is anonymized, your information will be kept private. You will not be identified in any publications resulting from this study. If you choose to participate, you are free to withdraw your permission at any time. You must do this in writing. Inform Dr. *Fouad Alshaban* on *falshaban@hbku.edu.qa* (phone #: 44541072) and let him know that you are withdrawing from the research study.

This study has been approved by the QBRI Institutional Review Board (ethics committee), If you wish to participate,

kindly check the box that applies to you: Select one *

- Yes, I consent to participate in this study
- No, I do not consent to participate in this study

Person filling this survey: Select one *

- Mother
- Father
- Other: (specify)___________________________

2- Nationality:* ___________________________

3- Diagnosis:* ___________________________

Does your child have any Comorbid conditions such as (ADHD, EPILEPSY, etc.)? Select one*

- Yes
- No

If you answered "yes" to the previous question, kindly specify which condition*: ______________________

4- Age of your son/daughter: **Select one***

- from 3 to 8
- from 9 to 13
- from 14 to 18
- Above 18

5- Gender of child: **Select one***

- Male
- Female

6- Number of children at home including your autistic child: **Please specify *** _________________________________

7- Do you have any other autistic children? Select one*

- Yes

- No

If you have more than one autistic child: specify how many*: ____

8- Father's Employment status: Select one*

- Employed
- Not Employed

9- Mother's Employment Status: Select one*

- Employed
- Not Employed

10- Are you currently working from home? Select one*

- Yes
- No

11- Has your family been reducing social contact as a result of COVID-19? Select one*

- Yes
- No
- To some extent

12- Has COVID-19 led to significant changes in your family's daily routine? Select one*

- Yes
- No

13- Who is the key person who provides support to your autistic child on a daily basis? Select one*

- You
- Your Spouse
- Other:

14- Do you have hired help at home (e.g. housekeeper, nanny, etc.)? Select one*

- Yes
- No

15- Name of center/school that your child was attending prior to the start of the home quarantine/physical distancing measures. If not receiving intervention or attending any school/center, write "none" *

____

Specify if your child was attending: Select one*

- Full-time
- Part-time
- Child was not receiving intervention

16- Was your child receiving in-home therapy? Select one*

- Yes

- No

If “yes”, how often? If no, write “none”: *

17- Was your child receiving additional therapeutic services outside the center? Select one*

- Yes
- No

If your answer on the previous question is “yes”, specify how often? If no, write “None” *

18- Since the start of the home quarantine/physical distancing measures, did you need healthcare services for your child? Select one*

- Yes
- No

If yes, for what type of services? If no, write “None” *

19- Since the start of the home quarantine/physical distancing measures, did you require emergency services for your child? Select one*

- Yes
- No

If yes, specify the reason. If no, write "None" *

20- Is your child abiding by the home quarantine/physical distancing measures? Select one*

- Yes
- No, I need to take him/her out

21- How did you explain the reason for the change in the daily routine (due to the home quarantine/physical distancing measures) to your child? (select all that is applicable) *

- Verbally
- With pictures
- Social story
- I couldn't explain it to my child
- Other:

22- How were COVID-19 preventative measures explained to your child? Select all that is possible *

- Verbally
- With pictures
- Social story
- I couldn't explain it to my child
- Other:____

23- Did you face any problems/issues with your child being at home all day? Select one*

- Yes
- No

If you responded yes, please explain further, and if you answered no, write "none" *

24- Is your child receiving any intervention sessions from a qualified specialist (who may be living with you) during the period of the home quarantine/physical distancing measures? Select one*

- Yes
- No

- To some extent

25- Since the start of the home quarantine/physical distancing measures, did you notice any regression in your child's previously gained skills? Select one*

- Yes
- No
- To some extent

If you answered "yes", kindly specify which skills you noticed a regression, and if you answered no, write "none": *

26- What are your child's favorite activities/games to do at home? Select all that is applicable: *

- Arts and crafts
- Card or board games (UNO, Monopoly, etc.)
- Electronics (Play station, iPad, television, mobile phone, etc.)
- All
- Other: specify

27- Is your child practicing any physical activity inside the home or outdoors (if you are living in a villa or a compound)? Select one*

- Yes
- No
- Sometimes
- We don't have outdoor space

28- Is your autistic child presently participating in online learning? Select one*

- Yes
- No
- To some extent

29- Did your child's sleeping schedule change since the start of the home quarantine/physical distancing measures? Select one*

- Yes
- No
- To some extent

30- Did you organize a daily schedule for your child since the start of the home quarantine/physical distancing measures? Select one*

- Yes
- No
- To some extent

31- Did your child exhibit any negative behaviors since the start of the home quarantine/physical distancing measures? Select one*

- Yes
- No

If you answered yes, specify which behaviors. If you answered No, write "none" *

32- Did you find difficulty receiving support from your extended family/friends/community during this time? Select one*

- Yes
- No
- To some extent

33- On a scale of 1-5, how would you rate your stress level during this time: Select one*

NOT STRESSED AT ALL	1	2	3	4	5	VERY STRESSED

34- On a scale of 1-4, how supported do you feel, whether from other family members, the community, etc. Select one *

No Support at all	1	2	3	4	Very Supported

35- In general, how would you rate your current health: Select one *

Excellent	1	2	3	4	5	Poor

36- Are you a member of a support or advocacy group for parents of autistic children? Select one *

- Yes
- No

37- Does your family presently have access to any online autism support (e.g. family support groups, therapeutic support, etc.)? Select one *

- Yes
- No

If you answered previous question "yes", specify the type of support, if "no" write (none): *

38- Is there any type of support that your family usually receives but cannot access in the meantime because of COVID-19? Select one *

- Yes
- No

If you answered previous question "yes", specify the type of support, if "no" write (none): *

39- Do you need support from Qatar Autism Society (QAS) and its partners during this time? Assistance such as phone/video consultation? A video conference to address your questions and any issues you may have, etc. Select one*

- Yes
- No

40- Do you have any suggestions for how we can further support you and your family? Select one*

- Yes
- No

If you answered "yes", kindly specify your suggestions, and if "no" write (none): *

41- Kindly share with us how you feel the pandemic related measures have affected you and your family, the main challenges you are facing, etc. *

In case we require further information from you, kindly provide us with your preferred mode of communication; phone number/email, if you don't prefer, write "none" *
